# Role of weight loss-induced prediabetes remission in the prevention of type 2 diabetes: time to improve diabetes prevention

**DOI:** 10.1007/s00125-024-06178-5

**Published:** 2024-05-23

**Authors:** Reiner Jumpertz von Schwartzenberg, Elsa Vazquez Arreola, Arvid Sandforth, Robert L. Hanson, Andreas L. Birkenfeld

**Affiliations:** 1 German Center for Diabetes Research (DZD), Neuherberg, Germany; 2 Department of Internal Medicine IV, Division of Diabetology, Endocrinology and Nephrology, Eberhard Karls University Tübingen, Tübingen, Germany; 3 Institute for Diabetes Research and Metabolic Diseases, Helmholtz Center Munich, Eberhard Karls University Tübingen, Tübingen, Germany; 4 Phoenix Epidemiology and Clinical Research Branch, National Institute of Diabetes and Digestive and Kidney Diseases, Phoenix, AZ USA; 5 Department of Diabetes, School of Cardiovascular and Metabolic Medicine & Sciences, King’s College London, London, UK

**Keywords:** Diabetes, DPP, Prediabetes, Prevention, Remission, Type 2 diabetes, Weight loss

*To the Editor:* By 2050, more than 1.3 billion people worldwide are expected to have diabetes, with the vast majority having type 2 diabetes [1]. Type 2 diabetes is associated with an increased risk of many chronic diseases, including micro- and macrovascular diseases, neurodegenerative disease and cancer, posing a huge burden on affected people and societies. Recent data show that more than 80% of those with type 2 diabetes will live in low- and middle-income countries and, thus, type 2 diabetes is becoming more and more a disease of inequity [1]. Prevention represents a key strategy for reducing the future incidence of type 2 diabetes and is therefore an urgent clinical need.

People with prediabetes (defined as fasting plasma glucose (FPG) ≥5.6 mmol/l, 2 h glucose during an OGTT ≥7.8 mmol/l or HbA_1c_ ≥39 mmol/mol (5.7%) but not meeting glycaemic criteria for type 2 diabetes [2]) have a lifetime risk of developing type 2 diabetes of 73%. In addition, prediabetes predisposes to diseases other than diabetes, particularly microvascular disease and CVD [3, 4]. We recently showed in a predefined post hoc analysis of the Prediabetes Lifestyle Intervention Study (PLIS) [5] using validation data from the US Diabetes Prevention Program (DPP; ClinicalTrials.gov registration no. NCT00004992) repository [5–7] that lifestyle-induced weight loss of >5% (including through dietary counselling and increased physical exercise) led to a remission of prediabetes to normal glucose regulation in 43% of participants, and provided these patients with a 73% relative reduction in the risk of developing type 2 diabetes compared to those not going into remission [5]. Remission rates increased with increasing weight loss. Thus, weight loss is an important driver of prediabetes remission and, in PLIS, an improvement in insulin sensitivity was critical for prediabetes resolution [5]. Previous analysis from the DPP showed that younger age and insulin secretion at baseline were predictive for remission [8].

In our analysis of PLIS and DPP participants [5], we defined remission as a return to normal glucose regulation, including normal FPG (<5.6 mmol/l), normal glucose tolerance (2 h post-load glucose <7.8 mmol/l) and HbA_1c_ <39 mmol/mol (<5.7%), at the end of the lifestyle intervention. Importantly, weight loss-induced prediabetes remission in PLIS participants not only reduced the relative risk (RR) of developing type 2 diabetes, but also was associated with lower renal albumin excretion and higher skin small vessel density as assessed by raster-scanning optoacoustic mesoscopy (RSOM) [5], suggesting improved small vessel integrity.

Current ADA standards of care for prevention and delay of diabetes recommend that people with prediabetes should lose ≥7% of their body weight; however, specific glucose targets are not recommended [9]. The 7% weight loss goal was chosen because it is feasible to achieve and maintain and is likely to reduce the risk of developing diabetes [9].

Here, we suggest that body weight loss and glycaemic remission goals should be considered together, as a combination of weight loss and remission of prediabetes provide the most effective protection against the development of type 2 diabetes. To support this notion, we used data from the DPP, which formed the basis of the current ADA standards of care for the prevention or delay of diabetes [9]. The DPP was a randomised multicentre clinical trial that studied the effects of an intensive lifestyle (ILS) intervention or metformin on the prevention or delay of type 2 diabetes in people with prediabetes [6, 8, 10]. Inclusion criteria were age ≥25 years, BMI ≥24 kg/m^2^ (≥22 kg/m^2^ for Asian participants) and a diagnosis of both impaired fasting glucose (6.1 mmol/l ≤ FPG ≤6.9 mmol/l) and impaired glucose tolerance (7.8 mmol/l ≤2 h post-load glucose ≤11.0 mmol/l). Participants were recruited between 31 July 1996 and 18 May 1999 from 27 clinical centres in the USA and were randomly assigned to receive the ILS intervention, metformin or a placebo. Data on participants’ sex were collected by self-report and the options provided were male or female. Participants in the ILS group received 16 one-to-one lessons covering diet, exercise and behaviour modification during the first 24 weeks, with advice to engage in moderate physical activity for at least 150 min per week. Diabetes was diagnosed using an annual OGTT or a semi-annual FPG test according to the ADA 1997 criteria [11]; diabetes diagnosis was confirmed by repeat testing [10]. The DPP cohort was representative of the US population at high risk of type 2 diabetes in terms of age, race/ethnicity and regional factors, and was characterised by more female participants [10]; minority groups constituted half of the study sample and the clinical centres were located across different regions of the USA [10]; and socioeconomic factors were not accounted for during recruitment.

Using data from the DPP, we compared the rate of incident type 2 diabetes over approximately 6 years in those with prediabetes who reached the guideline goal of ≥7% body weight loss during the lifestyle intervention but who did not go into remission of prediabetes (non-responders) with the rate in those who lost ≥7% of their body weight and additionally reached normal glucose regulation (responders), as defined above. This secondary analysis from the DPP repository included 480 participants randomised to the ILS intervention or placebo who lost ≥7% of their baseline body weight by year 1, had complete measurements of HbA_1c_, FPG and 2 h plasma glucose at baseline and year 1, and had follow-up data on diabetes diagnosis; participants assigned to metformin were not included. All DPP study participants gave written informed consent, and ethics approval for the study was provided by the Institutional Review Board of each clinical centre; these investigations were carried out in accordance with the Declaration of Helsinki as revised in 2013. Descriptive statistics for participants at baseline were calculated using frequency distributions for categorical variables. Continuous variables were summarised using arithmetic means and SDs or medians and IQRs and were compared using *t* tests or Wilcoxon tests as appropriate. Fisher’s exact tests were used to compare proportions of progressors and non-progressors to type 2 diabetes between groups and logistic regression models were used to determine if the probability of progression to diabetes differed between the groups when adjusting for treatment arm. Risk of progression to type 2 diabetes within the first 6 years of follow-up between groups was compared through RRs adjusted for treatment. Incidence of incident type 2 diabetes in all groups was visualised using Kaplan–Meier survival curves adjusted for treatment. Kaplan–Meier curves were compared using logrank tests. Descriptive characteristics of the included cohort are provided in Table 1.

**Table 1:** Descriptive characteristics of included DPP participants at baseline and 1 year

Characteristic	Baseline			1 year		
	Responders (*n*=114)	Non-responders (*n*=366)	*p* value^a^	Responders (*n*=114)	Non-responders (*n*=366)	*p* value^a^
Age (years), mean (SD)	49.8 (10.7)	53.2 (11.4)	0.005			
BMI (kg/m^2^)	33.0 (29.3, 38.4)	32.2 (28.9, 36.4)	0.101	28.0 (25.6, 32.8)	28.6 (25.4, 32.3)	0.604
Weight (kg)	94.3 (81.6, 108.8)	89.9 (78.0, 103.0)	0.027	80.3 (69.4, 95.8)	79.0 (69.2, 91.5)	0.279
Fasting glucose (mmol/l)	5.7 (5.5, 5.9)	5.8 (5.6, 6.2)	<0.001	5.2 (5.0, 5.3)	5.6 (5.3, 5.8)	<0.001
30 min OGTT glucose (mmol/l)	9.0 (8.3, 9.9)	9.4 (8.6, 10.4)	0.003	7.8 (6.9, 9.0)	8.8 (7.9, 9.8)	<0.001
120 min OGTT glucose (mmol/l)	8.7 (8.1, 9.7)	9.0 (8.4, 9.9)	0.005	6.0 (5.0, 6.7)	7.8 (6.5, 9.0)	<0.001
HbA_1c_ (mmol/mol)	36.6 (34.4, 38.8)	41.0 (38.8, 43.2)	<0.001	35.5 (32.2, 37.7)	39.9 (36.6, 42.1)	<0.001
HbA_1c_ (%)	5.5 (5.3, 5.7)	5.9 (5.7, 6.1)	<0.001	5.4 (5.1, 5.6)	5.8 (5.5, 6.0)	<0.001
Fasting insulin (pmol/l)	149.3 (111.1, 222.2)	166.7 (111.1, 229.2)	0.654	90.3 (62.5, 138.9)	104.2 (76.4, 145.8)	0.042
30 min OGTT insulin (pmol/l)	600.7 (364.6, 892.4)	583.4 (423.7, 847.3)	0.913	465.3 (284.8, 687.6)	472.3 (312.5, 652.8)	0.974
HOMA-B^b^	192.1 (144.3, 290.8)	195.1 (140.4, 266.7)	0.431	170.5 (108.3, 251.6)	151.1 (107.3, 204.9)	0.064
HOMA-IR^b^	5.5 (4.2, 8.1)	6.1 (4.2, 8.8)	0.367	2.9 (2.0, 4.7)	3.8 (2.6, 5.2)	0.001
Fasting insulin sensitivity index^c^	0.2 (0.1, 0.2)	0.2 (0.1, 0.2)	0.367	0.3 (0.2, 0.5)	0.3 (0.2, 0.4)	0.001
Proinsulin (pmol/l)	12.0 (8.0, 19.0)	14.0 (9.9, 21.0)	0.060	7.0 (4.0, 11.0)	8.3 (5.2, 13.0)	0.015
30 min corrected insulin response^d^	0.6 (0.4, 0.8)	0.5 (0.3, 0.7)	0.052	0.6 (0.4, 1.0)	0.5 (0.3, 0.7)	0.001

Data are median (IQR) unless indicated otherwise

^a^*p* values were derived from Wilcoxon tests

^b^HOMA-IR and HOMA-B were calculated as described previously [15]

^c^The fasting insulin sensitivity index was calculated as the reciprocal of HOMA-IR as described previously [16]

^d^The 30 min corrected insulin response was calculated as described previously [17]

Of the 480 participants who lost ≥7% of their body weight from baseline to year 1, 114 were responders (reaching normal glucose regulation at year 1) and 366 were non-responders (not reaching normal glucose regulation at year 1). There were 73 (64%) female participants in the responders group and 242 (66%) in the non-responders group. In total, 42 of 366 non-responders were diagnosed with type 2 diabetes by year 4 of follow-up compared with one of 114 responders. Responders had a significantly lower adjusted RR of progression to type 2 diabetes than non-responders over 6 years (RR 0.28, 95% CI 0.13, 0.64). By year 6 of follow-up, the proportion of people with incident type 2 diabetes was markedly lower among responders than non-responders (*p*=0.0002 by Fisher’s exact test). After adjusting for treatment arm, responders still had a significantly lower probability of progressing to type 2 diabetes than non-responders (OR 0.24, 95% CI 0.10, 0.58; *p*=0.0005).

Figure 1 shows the Kaplan–Meier diabetes-free survival curves for responders and non-responders adjusted for treatment arm. The probability of developing type 2 diabetes was lower in responders than non-responders from year 2 onwards (*p*=0.0005).

**Fig. 1:**
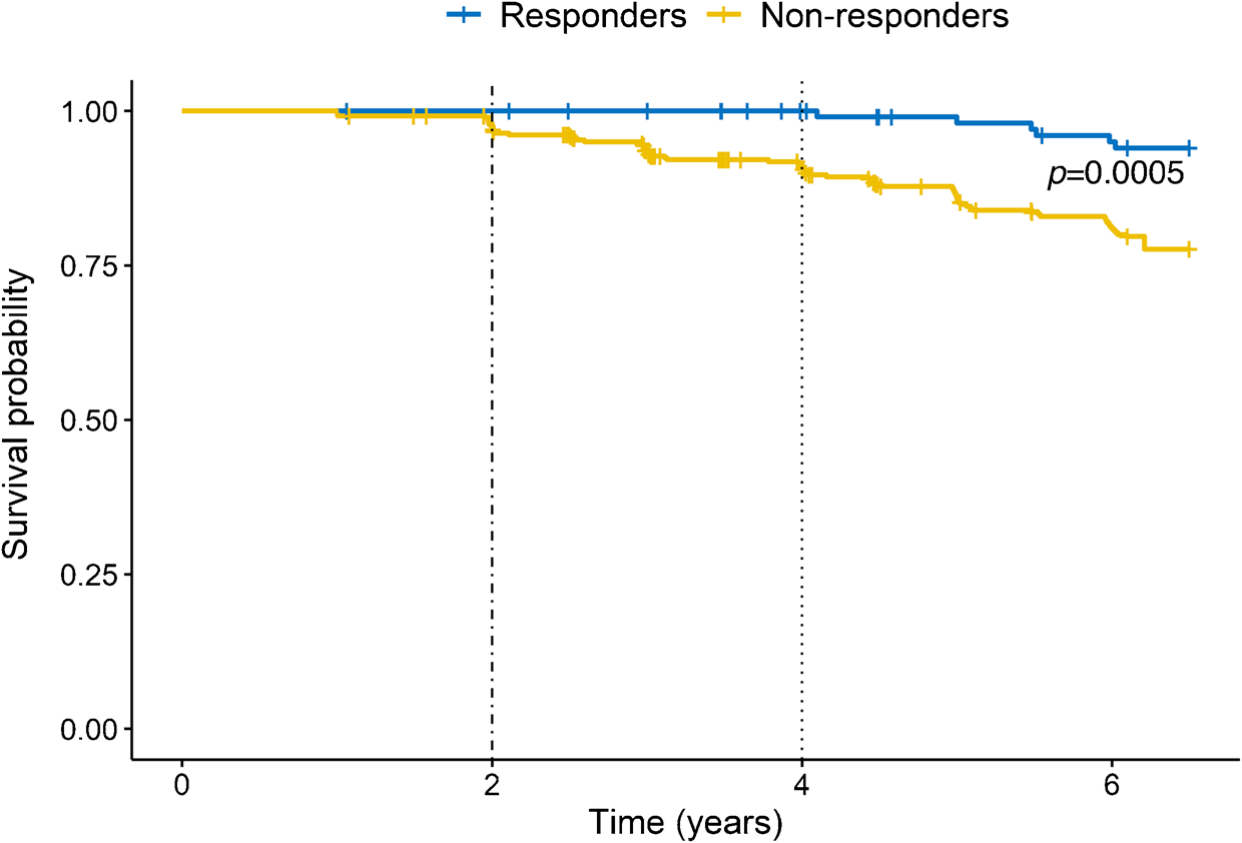
Kaplan–Meier curves for the probability of developing incident type 2 diabetes in responders and non-responders, adjusted for treatment arm. The probability of developing type 2 diabetes was lower in responders than non-responders from year 2 onwards (*p*=0.0005), leading to a continuous and progressive advantage over time for responders, that is, people who achieved remission of prediabetes after year 1. The dotted-dashed line at year 2 indicates the case-free interval for non-responders. The dotted line at year 4 indicates the case-free interval for responders

In summary, combining the recent ADA recommendation for people with prediabetes to lose ≥7% of their body weight [9] with remission from prediabetes (normal glucose regulation) reduced the RR of developing type 2 diabetes by 76% within 6 years; importantly, in the first 4 years of follow-up, there was only one incident diabetes case in the responders group, indicating that <1% of these participants developed type 2 diabetes after 4 years. As weight loss is a determining factor for the remission of prediabetes [7], we hypothesise that individuals with prediabetes who do not achieve remission (non-responders) after losing ≥7% of their body weight may benefit from continued weight loss until they reach their personal threshold [12, 13]. Other strategies such as increasing physical exercise levels should also be considered [14], as we have shown previously in the PLIS cohort that an ILS intervention is more effective at achieving remission than conventional lifestyle interventions [7]. Alternatively, if more weight loss is not possible, it would be important to sustain the weight loss achieved.

We conclude that adding glycaemic targets (i.e. normal glucose regulation) to weight loss targets in people with prediabetes provides a clear, measurable and reliable goal and is more effective at preventing type 2 diabetes than current recommendations. The concept of remission of prediabetes should be considered in future guidelines, as it has the potential to reduce the incidence and prevalence of type 2 diabetes worldwide and we hypothesise that it may be able to protect beta cell loss better than weight loss alone.

## Data Availability

The Diabetes Prevention Program (DPP) data reported here are available on request at the US National Institute of Diabetes and Digestive and Kidney Diseases Central Repository website (https://repository.niddk.nih.gov/).

## Abbreviations

DPP: Diabetes Prevention Program
FPG: Fasting plasma glucose
ILS: Intensive lifestyle
PLIS: Prediabetes Lifestyle Intervention Study
